# Candidate Gustatory Interneurons Modulating Feeding Behavior in the *Drosophila* Brain

**DOI:** 10.1371/journal.pbio.0030305

**Published:** 2005-08-30

**Authors:** Christoph Melcher, Michael J Pankratz

**Affiliations:** **1**Institut für Genetik, Forschungszentrum Karlsruhe, Karlsruhe, Germany; University of CambridgeUnited Kingdom

## Abstract

Feeding is a fundamental activity of all animals that can be regulated by internal energy status or external sensory signals. We have characterized a zinc finger transcription factor, *klumpfuss* *(klu),* which is required for food intake in *Drosophila* larvae. Microarray analysis indicates that expression of the neuropeptide gene *hugin* *(hug)* in the brain is altered in *klu* mutants and that *hug* itself is regulated by food signals. Neuroanatomical analysis demonstrates that *hug*-expressing neurons project axons to the pharyngeal muscles, to the central neuroendocrine organ, and to the higher brain centers, whereas *hug* dendrites are innervated by external gustatory receptor-expressing neurons, as well as by internal pharyngeal chemosensory organs. The use of tetanus toxin to block synaptic transmission of *hug* neurons results in alteration of food intake initiation, which is dependent on previous nutrient condition. Our results provide evidence that *hug* neurons function within a neural circuit that modulates taste-mediated feeding behavior.

## Abstract 2

Neuropeptide expressing neurons interconnecting taste input with higher brain centers modulate feeding behavior in Drosophila

## Introduction

All animals must be able to evaluate their nutrient requirement, as well as the nutrient supply offered by the environment, and translate the resulting information into appropriate behavioral responses. These can range from deciding to stop or continue feeding, or to look for alternate food sources. The nutrient signals can derive internally, reflecting the body's energy state and metabolic need, or through external sensory inputs, such as olfactory and gustatory signals. The sensory modalities further provide the basis for many types of higher brain functions, such as learning and memory. Feeding behavior, in turn, decisively influences almost all aspects of animal growth and reproduction. The role of the central nervous system (CNS) in integrating an animal's feeding behavior with sensory signals on the availability and quality of nutrients is, although undisputed, insufficiently understood [1].

*Drosophila* provides a genetically accessible system to study the molecular mechanisms that coordinate feeding behavior with sensory signals. This organism has an array of feeding characteristics that can be exploited for behavioral analysis, and insects in general have been used extensively as models for a wide range of behavioral and physiological studies [2,3]. In this context, the identification of genes encoding chemosensory receptors in *Drosophila* has provided a major impetus in understanding sensory signal transduction [4–8]. These genes have been broadly divided as encoding olfactory or gustatory receptors (ORs and GRs, respectively). Olfactory sensory neurons expressing specific ORs in the external mouth region project axons to distinct glomeruli of the antennal lobe [8–12]. Projection neurons then connect the antennal lobe to the mushroom body, where central processing of olfactory information occurs [13–15]. Gustatory sensory neurons are located not only in the external mouth region, but also internally in the pharynx; both types project to the subesophageal ganglion (SOG), a region implicated in feeding and taste response [8,16–18]. As compared with the antennal lobe, much less is known about the organization of the SOG—for example, whether it is also organized in glomerular structure. The neurons that connect the SOG to higher brain centers, in a manner analogous to the olfactory projection neurons, have also not been identified.

In both olfactory and gustatory cases, the knowledge is even sparser concerning the identity of interneurons that act between the sensory neurons and motor or neuroendocrine outputs and how they might influence feeding behavior. Studies in different insects have shown that various parts of the CNS are interconnected with the neuroendocrine organs and the enteric (stomatogastric) nervous system, which innervates the feeding apparatus [19,20]. The mouth parts have also been shown to be innervated by nerves from the SOG [21]. Nevertheless, an integrated map of the neurons comprising these circuits and their function in mediating a behavioral response has been lacking.

We have previously identified a gene, *pumpless* *(ppl),* that is required for food intake behavior in the *Drosophila* larvae [22]. It encodes a subunit of the glycine cleavage system and is expressed exclusively in the fat body. Although not feeding, *ppl* mutant larvae do not show characteristics of starving larvae, as assayed both by molecular markers and behavioral characteristics; furthermore, feeding high levels of amino acids can phenocopy several aspects of the *ppl* feeding phenotype. These observations led to a model in which amino acid-dependent signals from the fat body to the brain can signal cessation of feeding. In this study, we characterize another mutant, *klumpfuss* *(klu),* with a phenotype very similar to *ppl* mutants. Through microarray analysis, we identified the neuropeptide gene *hugin (hug)* as being deregulated in *klu* mutants. *hug* is expressed in a small assembly of about 20 neurons in the SOG. Connectivity mapping and behavioral studies suggest that *hug*-expressing neurons function in a neural circuitry in the brain that modulates chemosensory signal-dependent feeding behavior.

## Results

### Molecular Characterization of Larval Mutant Defective in Feeding Behavior

**Figure 1:**
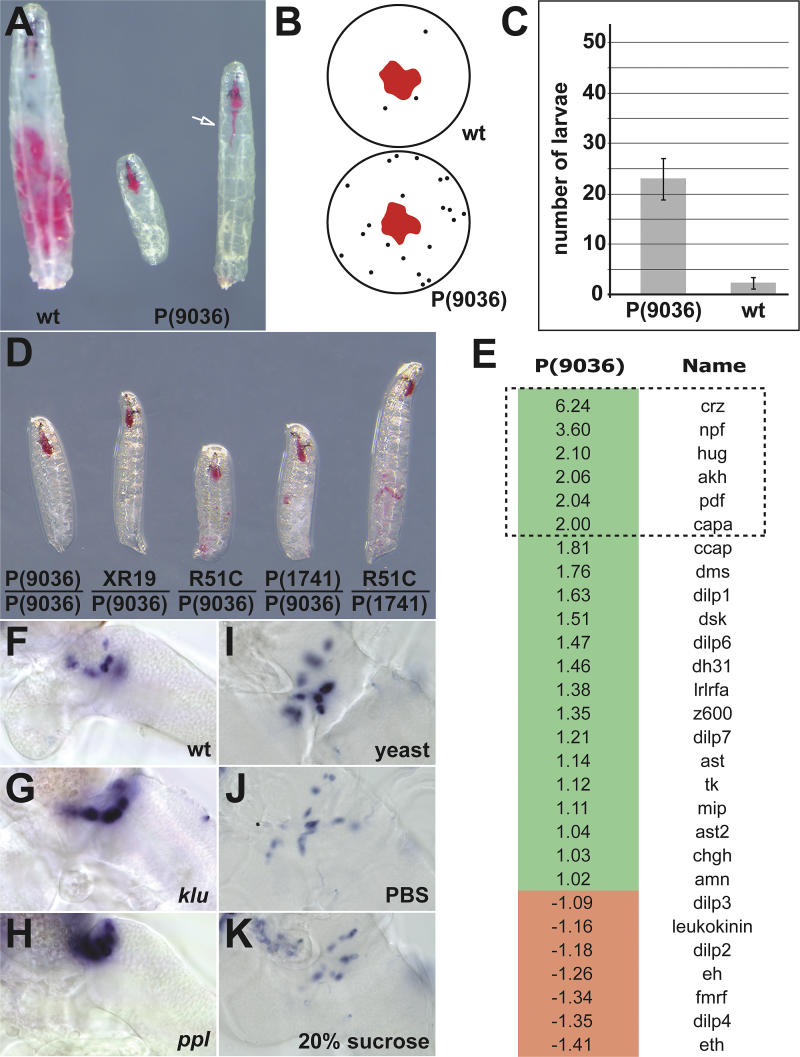
Phenotypic Characterization and Expression Analysis of P(9036) Mutants (A) P(9036)-mutant larvae show feeding and growth defects as compared to wild-type (wt) controls. In wild-type, red food fills the gut, whereas it accumulates in the pharynx of P(9036) mutants. Physically, the food can pass the mutant pharynx (see arrow). (B) P(9036) mutants show a wandering behavior, in which they leave the food source (depicted in a schematic snapshot drawing; dots represent larvae outside the food source, which is shown in red). (C) Quantification of the P(9036) wandering phenotype shows that from 50 individuals, 23 ± 4 mutants can be found outside the food source, in contrast to only 2 ± 1 for the wild-type control at a given time point (*n* = 8 time points). (D) The known *klu* alleles *klu*XR19, *klu*R51C, and P(1741) fail to complement P(9036), and trans-heterozygous larvae display the feeding phenotype. (E) Microarray analysis of P(9036) revealed several neuropeptide genes as being deregulated in P(9036) mutants compared to wild-type controls (×-fold changes in P(9036) are listed; genes with more than 2-fold change are boxed). (F–H) Semi-quantitative in situ hybridizations to first instar larval brains verify the upregulation of the neuropeptide gene *hug* in P(9036) *klu* mutants in comparison to wild-type controls, and show an upregulation of *hug* in *ppl* feeding mutants as well. The expression of *hug* is in the SOG. Anterior points to the upper left corner; ventral views are shown. (I–K) Semi-quantitative in situ hybridizations to late second instar larval brains demonstrate a downregulation of *hug* in wild-type under starvation (PBS) and sugar conditions (20% sucrose), in comparison to normal feeding condition (yeast). Anterior points to the upper left corner; ventral views are shown.

In a screen for *Drosophila* mutant larvae defective in feeding, we identified the P-element line P(9036). These animals fail to pump food from the pharynx into the esophagus (Figure 1A), which is not due to a morphological block in the esophagus. The failure to feed is also not due to a general illness of the animal or global locomotory defects, because they can move around with the same vigor as wild-type or heterozygote siblings. P(9036) larvae also display wandering-like behavior, in which they move away from the food (Figure 1B and 1C). During this wandering-like phase, P(9036*)* larvae move about with food lodged in their pharynx, further supporting the view that the feeding defect is not due to a general body movement defect. Wandering behavior is observed in wild-type larvae when they stop feeding and move away from food shortly before pupariation [23]. These feeding behavior defects have also been observed for *ppl* mutants [22]. *ppl* encodes an amino acid catabolizing enzyme that is expressed exclusively in the fat body, an organ analogous to the vertebrate liver. Thus, P(9036) and *ppl* mutants, as immature first instar larvae, display feeding behaviors characteristic of sated, full-grown, third instar larvae. We characterized the gene corresponding to P(9036) and found it to be *klu,* a zinc finger protein-encoding gene that is expressed specifically in the developing nervous system [24,25]. P(9036) fails to complement the lethality of all *klu* alleles tested, and trans-heterozygotes also show the characteristic feeding defect (Figure 1D).

### The Neuropeptide Gene *hug* Expression is Altered in *klu* Mutants and in Amino Acid-Deficient Conditions

**Figure 2:**
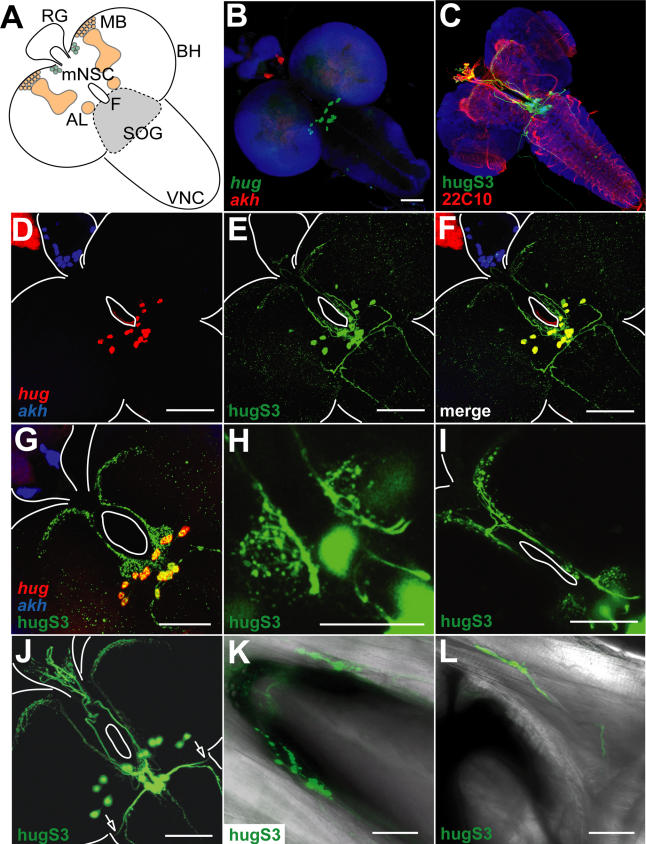
Neuroanatomical Analysis of *hug* Expression and Neuronal Projection Patterns in Larvae (A) A schematic drawing of the *Drosophila* larval CNS, showing the relative positions of the two brain hemispheres (BH) and the VNC, with the neuroendocrine ring gland (RG) located dorsoanterior to the CNS. The esophagus goes through the brain, and we have termed the hole through which the esophagus passes as the foramen (F). Positions of the SOG, the larval antennal lobes (AL), the mushroom bodies (MB), and the median neurosecretory cells (mNSC) are depicted. (B) *hug* expression (shown in green) is restricted to the SOG. *adipokinetic hormone* (*akh*, shown in red) serves as a ring gland marker; the CNS is false-colored in blue. (C) Detection of marker gene expression under *hug* promoter-Gal4 (hugS3) construct (shown in green) labels *hug* cell bodies and neuronal projections relative to the neuropil (22C10, shown in red) and nuclei of post-mitotic neurons (elav, shown in blue). (D–F) The endogenous *hug* expression pattern (shown in red in [D]) is reproduced by the hugS3 expression pattern (shown in [E]). Note double-positive cell bodies in (F). *akh* serves as ring gland marker; CNS, ring gland, and foramen are outlined. (G and H) Immunofluorescent (G) as well as direct (H) detection of GFP expressed under the *hug* promoter-Ga14 construct reveals putative *hug* dendrites dorsoanterior of the *hug* cell bodies, where spherical structures are innervated. The *hug* cell bodies in the depicted blow-up of SOG region are out of focus in (H). (I) *hug* axons innervating the protocerebrum cross the midline, showing ipsilateral and contralateral innervation of mushroom body region. (J–L) Expression of eGFP under hugS3 shows *hug* axons leaving the SOG laterally (see arrows). These can be followed to the cephalopharyngeal complex of the larvae (K and L) onto the pharyngeal muscles (axons shown in green overlayed with transmission light picture; note the mouth hook apparatus in black). Dorsal (K) and lateral views (L) are shown, anterior points to upper left corner. All fluorescence images in this and subsequent figures are Z-stack projections, and all scales bars are 50 μm, unless otherwise noted.

To study the central control process that could underlie the feeding defect of *klu* mutants, we performed microarray analysis of *klu* mutants with a focus on neuropeptide genes. We reasoned that their expression patterns in the brain would be specific enough for analysis at single-cell resolution. Furthermore, neuropeptides have been shown to influence food intake in different organisms, including mammals [1]. RNA from *klu* mutant larvae and wild-type larvae were isolated and hybridized to three Affymetrix chips each and compared. Figure 1E lists *Drosophila* neuropeptide genes and their expression profile in *klu* mutants, relative to wild-type first instar larvae. We then performed in situ hybridizations on wild-type larval brains with the six highest upregulated genes. We decided to focus our efforts on *hug* because it had the most specific expression pattern in the larval brain. While all others showed staining in different parts of the brain or in the ventral nerve cord (VNC) (unpublished data), *hug* showed staining in only a cluster of about 20 cells in the SOG of the larval brain, with no staining anywhere else (Figures 1F and 2). Its expression in embryos is also highly restricted in the brain [26]. *hug* encodes a prepropeptide capable of generating at least two neuropeptides, Drm-PK2 and hug-γ. The former encodes a myostimulatory peptide while the latter shows homology to ecdysis-triggering hormone-1, ETH-1 [26]. Both can activate a G-protein–coupled receptor belonging to the vertebrate neuromedin U group [27]. A *hug* homolog is also found in *Anopheles* *gambiae* [28].

To confirm the microarray data, we performed semi-quantitative in situ hybridization in wild-type and *klu* mutant larval brains. *hug* is upregulated in *klu* mutants (Figure 1F and 1G). We then investigated whether *hug* expression is also regulated in *ppl* larvae, which display a similar feeding defect as *klu*. There is also an upregulation of *hug* in *ppl* mutants (Figure 1H). We next investigated whether *hug* expression is regulated by different nutrient signals. We therefore placed wild-type larvae in starvation and sugar conditions (that is, both being amino acid-deficient diets) and monitored *hug* expression. *hug* was downregulated in both conditions (Figure 1I, 1J, and 1K), indicating a response to nutrient signals distinct from simple lack of energy. As *hug* is upregulated in *klu* and in *ppl* mutants, both of which do not feed and wander about, a higher *hug* level correlates with decrease of food intake and food-seeking behavior. For *hug* downregulation under starvation and sugar conditions, a lower *hug* level correlates with increased food-seeking behavior since *Drosophila* larvae become hyperactive and disperse when food is removed.

### *hug-*Expressing Neurons Project to the Ring Gland, the Pharyngeal Muscles, and the Protocerebrum

As mentioned above, *hug* is expressed specifically in a small group of neurons in the SOG (Figure 2A and 2B). To gain insight into the physiological processes that *hug*-expressing neurons (referred to as *hug* neurons) could be involved in, we wanted to determine their connectivity pattern. Therefore, we constructed a *hug* promoter-Gal4 line (hugS3) in order to express different versions of green fluorescent protein (GFP) marker genes for neuroanatomical studies. This approach revealed *hug* neuron projection to the ring gland (Figure 2C). The ring gland, as the master neuroendocrine organ of *Drosophila* larvae, controls metabolism and growth. For example, median neurosecretory cells of the pars intercerebralis that express *Drosophila* insulin like peptides (dilps), also project to the ring gland, whereas adipokinetic hormone (akh), thought to be a glucagon homolog, is produced by the ring gland [29–31].

In addition to the ring gland, we also observed *hug* neuron projection to the protocerebrum, near the median neurosecretory cells and the mushroom bodies (Figure 2D–2F), which comprise the center for olfactory learning and memory [13,32]. The axons projecting to the protocerebrum also cross at the midline just above the foramen (Figure 2I). We also noticed an intriguing glomerular-like structure of what are most likely *hug* dendrites in the SOG, just dorsoanterior to the *hug* cell bodies (Figure 2G and 2H). Singh [17] has described glomerular organization in the SOG of adult *Drosophila* that relays gustatory information. Such a glomerular organization has not been previously recognized for the larval SOG, but it would be analogous to the glomerular organization in the antennal lobes that relay olfactory information.

Strikingly, there is also projection of *hug* neurons to the pharyngeal muscles (Figure 2J, 2K, and 2L), which pump food into the mouth atrium. These arise from axons that leave the brain (Figure 2J) and project anteriorly along each side of the dorsal pharyngeal muscles, and terminate near the anterior end of the pharynx (Figures 2K, 2L, and 4). There has been no previous case of identified neurons in the larval SOG that project to motor outputs. At this point, we do not know whether the pharyngeal muscles are actually innervated by these axons. Taken together, these results demonstrate that *hug* neurons in the larvae project to key organs regulating feeding and growth—namely, the pharynx and the ring gland—as well as to higher brain centers.

### *hug* Dendrites Innervate GR-Expressing Sensory Organs and Chemosensory Organs of the Pharynx

**Figure 3:**
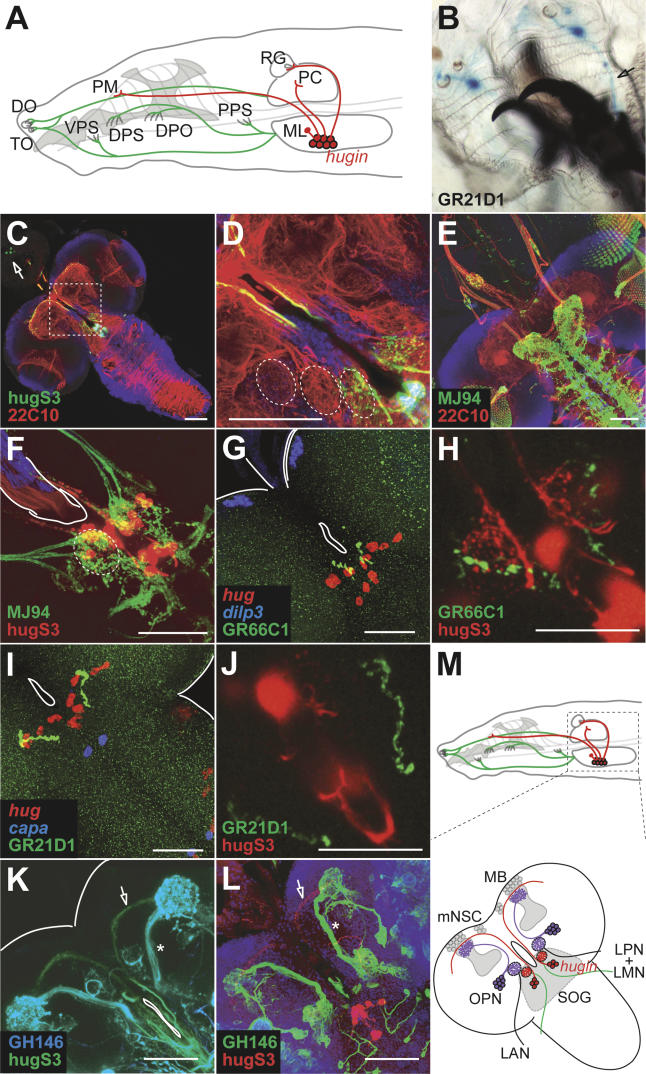
hug Neurons Receive Gustatory Input (A) Schematic drawing of the head region of a *Drosophila* larva with external as well as internal chemosensory neurons in antennomaxillary complex and internal mouth region innervating the larval CNS (depicted in green). *hug* neurons in the SOG (shown in red) project to the ring gland (RG), pharyngeal muscle (PM) region, and the protocerebrum (PC). Relative positions of external chemosensory sensillae (dorsal organ [do] and terminal organ [to]), internal chemosensory sensillae (ventral pharyngeal sense organ [vps], dorsal pharyngeal sense organ [dps], dorsal pharyngeal organ [dpo], and posterior pharyngeal sense organ [pps]), and major projections to the CNS are shown. (B) GR21D1-positive sensory neurons (shown by X-Gal staining) in the dorsal organ project axons to the CNS (see arrow). (C and D) Optical section through median CNS (composed of ten confocal 1-μm sections) shows *hug* arborizations in the SOG and mushroom body region (shown in green) relative to general neuropil (red) and cortical (DNA marker Draq5, blue) landmarks. Note the labeling of corpora allata cells in the ring gland (arrow; see Materials and Methods and Figure S2). Boxed area is shown at higher magnification in (D), revealing spherically organized neuropil regions lateral to foramen (partially outlined). (E) Expression of nSyb-GFP under MJ94 enhancer trap construct labels axon terminals of internal gustatory sensory neurons (shown in green), innervating SOG and VNC. (F) Close-up of SOG region shows co-localization of spherically organized axon terminals of MJ94 positive gustatory neurons (green) and *hug* neuronal arborizations (red). (G) Axon terminals of GR66C1-positive chemosensory neurons (shown in green) can be detected in the vicinity of *hug* cell bodies (shown in red). *dilp3* staining (blue) serves as morphological landmark. (H) Optical section (composed of ten confocal 1-μm sections) containing the GR66C1 axon terminals (shown in green) also include the spherical *hug* arborizations. *hug* cell bodies are out of focus in (H); a close-up of the SOG is depicted. (I and J) Axon terminals of GR21D1 positive chemosensory neurons (shown in green) also project to the vicinity of *hug* cell bodies (shown in red in [I]), but the optical section comprising these terminals does not contain the *hug* arborizations (note the absence of spherical *hug* dendrites in [J] as compared to [H]). (K–M) Comparison of axon tracts used by olfactory projection neurons, which can be labeled by enhancer trap line GH146 (shown by GFP real color in [K] and in green in [L], marked by asterisks), with those used by *hug* neurons (shown by YFP real color in [K] and in red in [L], marked by arrows), indicate that *hug* neurons are distinct from olfactory projection neurons (OPN). These differences are summarized in (M). LAN, larval antennal nerve; LMN, larval maxillary nerve; LPN, larval pharyngeal nerve.

The projection of *hug* neurons to the mushroom body region, together with the fact that *hug* is expressed specifically in the SOG, which relays gustatory information, suggested that *hug* neurons could be involved in mediating chemosensory signals. Therefore, we investigated whether *hug* neurons receive direct input from the chemosensory organs (Figure 3A–3D). It has been demonstrated that sensory organs in the larval head that express ORs or GRs send their axons either to the antennal lobe or the SOG [8] (Figure 3A and 3B). Recently, an enhancer trap line MJ94 was used to label putative chemosensory organs of the internal pharynx [18]. As internal pharyngeal sensory organs are good candidates for transducing gustatory signals, we wondered if these sensory organs terminate at *hug* dendrites. As shown in Figure 3E and 3F, they indeed terminate in the contact region of *hug* dendrites.

To further test this, we checked to see if chemosensory neurons that express specific GRs also project to *hug* dendrites. For example, it has been shown that GR66C1-positive neurons project to the SOG, whereas GR21D1-positive neurons project to the antennal lobe [8]. To see if these sensory projections terminate at or near *hug* neurons, we first performed staining of GR66C1- and GR21D1-positive axon terminals with *hug* in situ hybridization. GR66C1 receptor neurons indeed project to the vicinity of *hug*-expressing cells (Figure 3G). To see if these axons may potentially make synaptic contacts with *hug* dendrites, we used a *hug* promoter–yellow fluorescent protein (YFP) line (in which YFP was placed directly under the *hug* promoter), in combination with GR promoters driving nSyb-GFP [8,33], thus allowing simultaneous visualization of GR axon terminals and *hug* dendrites. As shown in Figure 3H, GR66C1-positive neurons project to the glomerular-like SOG region contacted by *hug* dendrites. GR21D1-positive neurons also project near the *hug* cells (Figure 3I), but by contrast to GR66C1-positive neurons, do not contact *hug* dendrites (Figure 3J). Rather, they terminate dorsoanterior to the *hug* dendrites, where the antennal lobes are located [8]. Taken together, these results suggested that *hug* neurons may act as second-order interneurons that relay gustatory information.

To further distinguish the relationship between *hug* neurons and the olfactory or gustatory systems, we determined whether *hug* neurons share the same axon tracts to the mushroom bodies as the second-order neurons that relay olfactory sensory input. The dendrites of these olfactory projection neurons underlie the glomerular structure of the antennal lobes and vertically transduce olfactory information for processing to the mushroom bodies. These projections can be visualized by the enhancer trap line GH146 [14]. As shown in Figure 3K and 3L, the axon projections of *hug* neurons are distinct from olfactory projection neurons: *hug* neurons project to a more dorsomedial region in the protocerebrum than olfactory projection neurons, and they use different axon tracts. These results essentially rule out *hug* neurons being olfactory projection neurons. Projection neurons transducing gustatory signals to higher brain centers have not yet been identified. In this context, *hug* neurons could act as gustatory projection neurons that connect gustatory sensory neurons via SOG with the protocerebrum (Figure 3M).

### Subpopulation of *hug* Neurons Project to Distinct Targets

**Figure 4:**
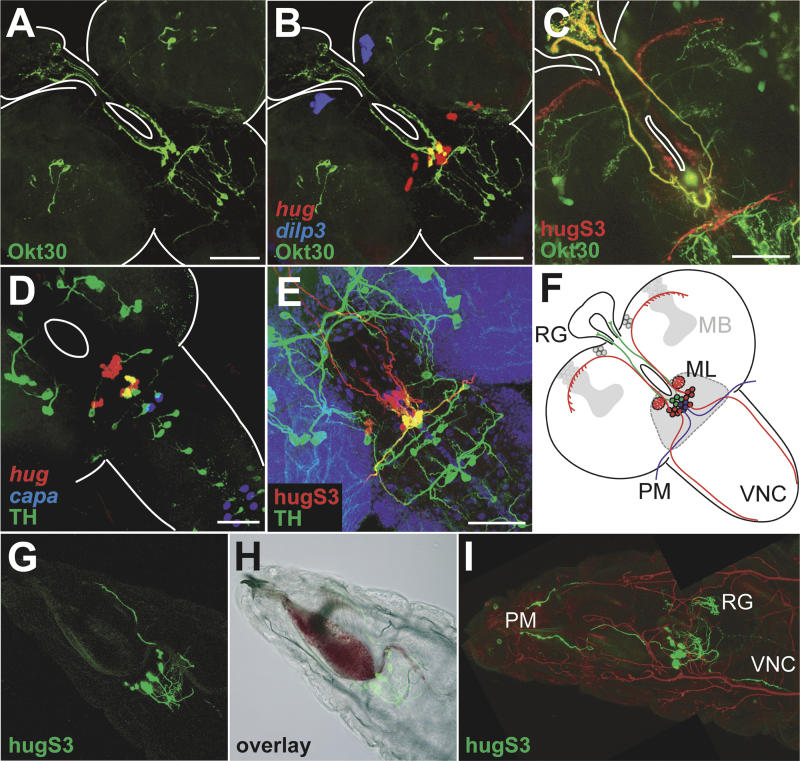
Subpopulations of *hug* Neurons Innervate Distinct Targets (A and B) Enhancer trap line Okt30 (shown in green) labels SOG neurons projecting to ring gland. Okt30 expression pattern colocalizes with *hug* expression (shown in red in [B]). There are four double positive cells in (B). *dilp3* staining (blue) serves as morphological landmark. (C) Direct detection and false colorization of GFP expressed in Okt30 positive cells (green) and YFP expressed under *hug* promoter (red) reveals only the axons to ring gland as double positive. (D) TH promoter construct labels dopaminergic CNS neurons (shown in green). Four TH-positive SOG neurons also express *hug* (shown in red). *capa* staining (blue) serves as morphological landmark. (E) Combination of projection patterns of TH positive cells (green) with *hug* cells (red) reveals the axons innervating the pharyngeal muscles as the only double positive ones. (F) Schematic summary of (A–E) showing distinct subpopulations of *hug* neurons projecting to the ring gland only (green), pharynx only (blue), and the remaining targets (red). (G–I) Projection pattern of *hug* is unaffected in *klu* mutants. Direct detection and false coloring of *hug* promoter-YFP (shown in green) in the *klu* background reveals the pharyngeal muscles (PM), the ring gland (RG), and the VNC as being targeted in feeding mutants. Trachea are false-colored (red) in composite figure (I).

We have also noticed a difference in the projection specificity among the *hug* neurons. A series of enhancer trap lines have been isolated that label cells projecting their axons to the ring gland [34], one of which (Okt30) is co-expressed with *hug* (Figure 4A and 4B). When we use our *hug* promoter-YFP line (to distinguish it from GFP reporter constructs) in combination with the Okt30 ring gland enhancer trap line, we find that a distinct set of *hug* neurons project to only the ring gland and not to the protocerebrum, the pharynx, or the ventral cord (Figure 4C).

Another subpopulation of *hug* neurons is revealed by using the TH-Gal4 line. This drives reporter gene expression under the promoter of tyrosine hydroxylase (TH), a key enzyme in dopamine synthesis [35]. As shown in Figure 4D, specific *hug* neurons express TH-Gal4 reporter gene, indicating that a subset of *hug* neurons might be dopaminergic. When TH-Gal4-driven lacZ is used in combination with *hug* promoter-YFP, we observe that TH-positive *hug* neurons project to only the pharynx and not to the protocerebrum, the ring gland, or the VNC (Figure 4E). These results indicate that at least three distinct subpopulations of *hug* neurons exist: those projecting to only the ring gland, those projecting to only the pharyngeal muscles, and those projecting to the protocerebrum and/or the VNC (Figure 4F). The distinct target specificity suggests differences in the function of the *hug* subpopulations. In the honeybee *Apis,* the subesophageal-calycal tract neurons are located in the SOG, send axons to the protocerebrum, and receive input from the sensory neurons of the proboscis; these neurons are thought to transduce gustatory information [36]. Some of the *hug* neurons could act similarly to these honeybee neurons. Based on the connectivity map of the *hug* neurons, we also wondered if the global targeting of these neurons was altered in *klu* mutant larvae. Therefore, we crossed the *hug* promoter-YFP construct into *klu* mutant background (Figures 4G and 4H). Although we cannot rule out subtle local differences, the basic connectivity pattern is retained in the mutants (Figure 4I).

### *hug* Neuron Connectivity Pattern Is Similar in Larvae and Adults

**Figure 5:**
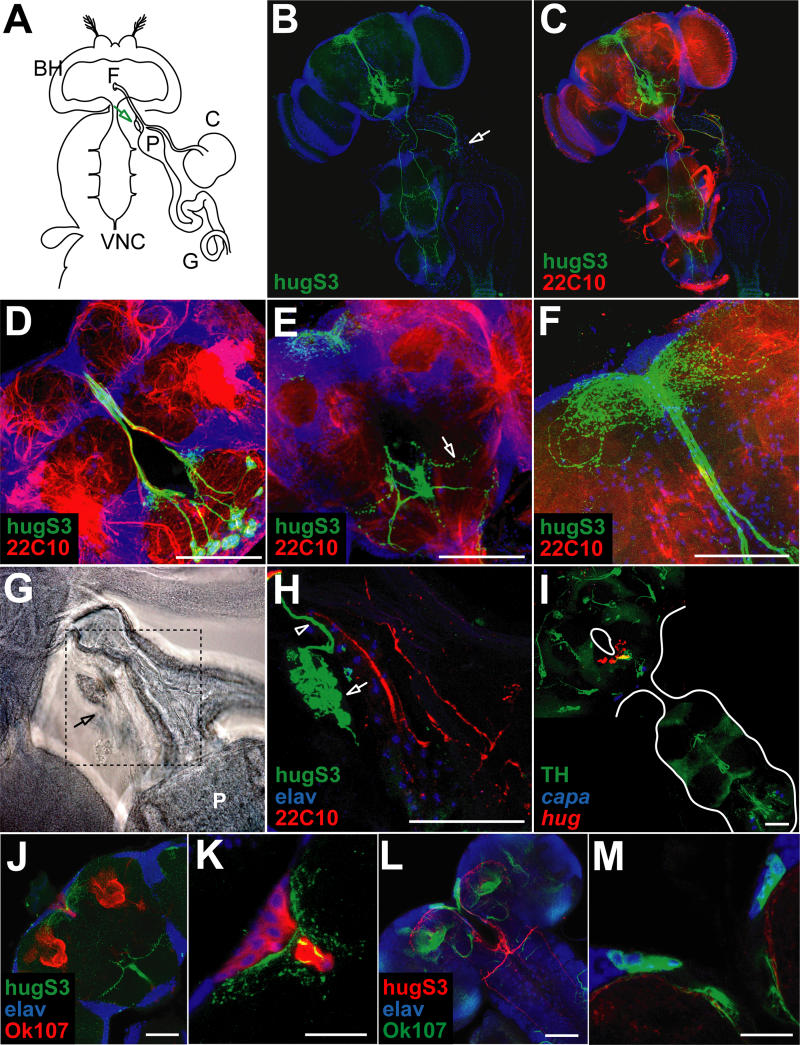
Neuroanatomical Analysis of *hug* Interneurons in Adults (A) Relative positions of adult CNS composed of brain hemispheres (BH) and VNC, as well as esophagus passing the brain through the foramen (F), proventriculus (P), crop (C), and gut (G) are depicted schematically. The neuroendocrine CC/CA complex is located on top of the proventriculus (arrow). (B) Localization of *hug* neurons and projections in adult CNS relative to nuclear marker. Note projections to the protocerebrum (top of the head), VNC, and CC/CA (arrow). (C) *hug* neuronal projections relative to general neuropil marker (22c10, shown in red). (D–F) Close-ups of different optical sections (composed of confocal 1- to 2.5-μm sections) show spherical *hug* arborizations in median SOG region (green in [D]), arborizations in lateral SOG region (green in [E], arrow) and in protocerebrum (green in [F]). (G) Transmission light image of CC/CA complex (arrow) on top of proventriculus (P). (H) Immunofluorescent close-up of the area boxed in (G). Nuclei of elav positive CC cells (blue) and of CA (green, see arrow), *hug* axon terminals (green, see arrowhead). (I) TH positive SOG neurons (green) co-express *hug* (red); *capa* staining serves as morphological landmark. (J and K) Different optical sections (composed of confocal 1- to 2.5-mm sections) show *hug* (green) projections above the mushroom bodies and adjacent to the median neurosecretory cells marked by OK107 (mushroom body and median neurosecretory cell marker, red); *elav* marker, blue. (K) Close-up of the region in (J), showing the medial neurosecretory cells. Scale bars equal 20 μ. (L and M) Different optical sections (composed of confocal 1-to 2.5-mm sections) showing OK107 (green) and *hug* (red) in larva. (M) Close-up of the median neurosecretory cell region. Scale bars equal 20 μ.

To see if *hug* neurons might also have a function in the adults, we determined the connectivity pattern in adult animals. There are some noticeable morphological differences in the feeding apparatus and neuroendocrine organs between adults and larvae (Figure 5A). One is the presence of the crop in the adult but not in the larva. The crop is a food storage organ, and its absence in the larvae most likely reflects a difference in the feeding habits; whereas adults are intermittent feeders, the larvae feed continuously. Another is the relocation of the neuroendocrine organs. The corpora cardiaca/corpora allata (CC/CA) complex, which comprises part of the ring gland in the larvae, is located right above the proventriculus in the adults, at the junction between the gut and the crop. This is in contrast to the larvae, where it is located on top of the brain hemispheres. Monitoring hugS3 expression in adults, we observe axon projections to the protocerebrum, the CC/CA complex, and the ventral cord (Figure 5B–5H). A subpopulation of *hug* neurons may also be dopaminergic, as in the larvae (Figure 5I). To further characterize the projections to the protocerebrum, we used the OK107 enhancer trap Gal4 line [37] together with *hug* promoter-YFP. These stainings indicate that *hug* axons traverse along the median neurosecretory cells in the pars intercerebralis, and terminate near the mushroom bodies (Figure 5J and 5K). A similar pattern is observed in the larvae (Figure 5L and 5M). The precise targets of *hug* neurons projecting to the protocerebrum remain to be determined. Taken together, despite the morphological differences, the connectivity pattern of *hug* neurons is remarkably similar between larvae and adults.

### Blocking Synaptic Transmission of *hug* Neurons Alters Food Intake Behavior

**Figure 6:**
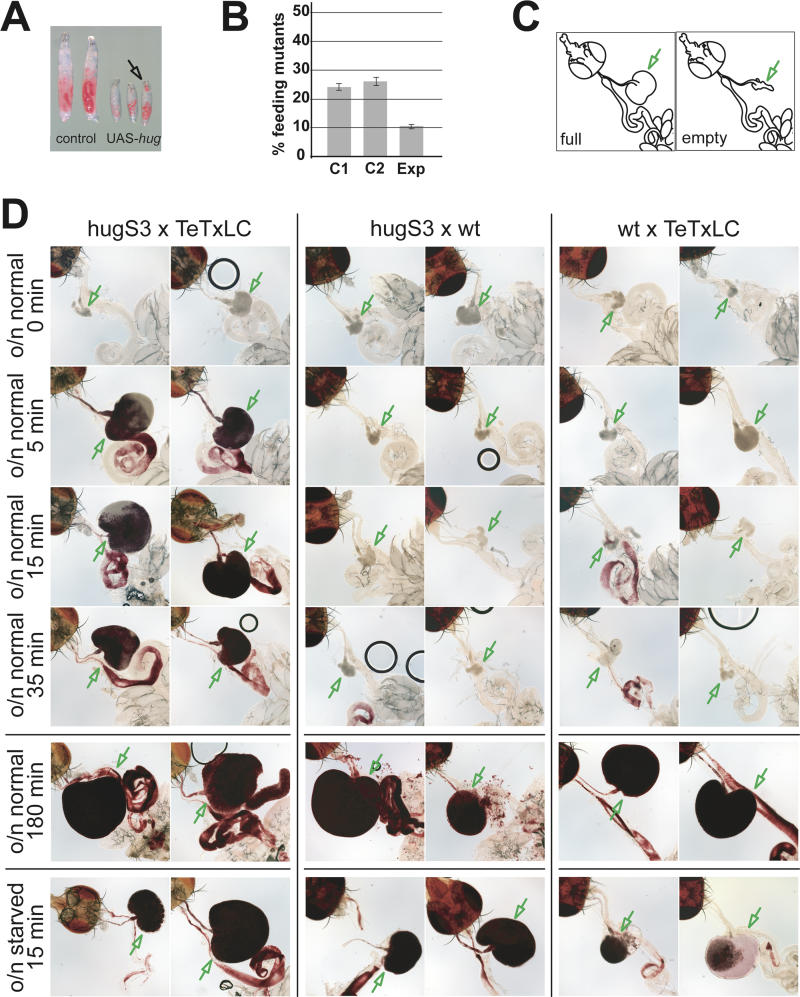
Overexpression of *hug* and Blocking *hug* Synaptic Transmission Causes Feeding Phenotypes (A) Overexpression of *hug* neuropeptide gene under ubiquitous promoter leads to reduced feeding and growth (compare size of UAS-*hug* larvae and controls of same age) as well as to larval lethality. Note the individual phenocopying the *klu* feeding phenotype (arrow). (B) Partial rescue of *klu* feeding defect by blocking *hug* neuronal activity. *n*= 5; error bars represent standard deviation; see Materials and Methods for details. (C) Schematic of adult internal morphology with proventriculus, crop, and gut. Crop is depicted in full and empty state. (D) Feeding behavior of adult flies monitored by the presence of red food in the midgut and crop (marked by arrows). Starting from the same feeding status (empty crop at time point 0 min), experimental flies expressing TeTxLC under hugS3 construct initiate uptake of red food immediately (notice large amount of red food in crop and gut after 5 min) when confronted with red yeast paste after feeding on standard fly food overnight (overnight normal). In contrast, control flies (hugS3 flies and TeTxLC flies crossed with wild-type) initiate food uptake after 15–35 min (notice traces of red food in midgut) when confronted with red yeast paste. The same feeding status is detected in experimental as well as in control flies after long feeding period on red yeast (overnight normal, 180 min). Overnight starvation equalizes feeding behavior when flies are confronted with red yeast paste for 15 min (notice same amount of red food in all cases at overnight starvation 15 min). Two representative samples of each time point and genotype are displayed. Two independent experiments were carried out, and for each experimental set ten individuals were taken out randomly and dissected. From 20 total flies, 16 ± 2 showed the phenotype displayed here o/n, overnight.

Based on the connectivity map of *hug* neurons and the alteration in *hug* expression under different nutrient and feeding conditions, we initiated a series of experiments to explore the role of *hug* in regulating feeding. As *hug* mutants have not yet been identified, we tested the effects of overexpressing *hug* in the larvae. We first used hugS3 to drive *hug* expression but did not observe any phenotype (unpublished data). This is most likely because using an endogenous promoter does not result in high enough overexpression of *hug* in cells that already express physiological levels of *hug*. We therefore used a strong ubiquitous promoter (tubulin-gal4). There was a strong reduction in growth (Figure 6A), with no larvae surviving to pupal stage; we also observed defects in food intake, although not to the same strong degree as with *klu* mutants (Figure 6A). This is consistent with the view outlined earlier that high *hug* levels correlate with decreased food intake.

In order to gain further information on the function of *hug* neurons, we then blocked synaptic transmission in these cells using tetanus toxin light chain (TeTxLC) [38]. We first carried out the experiments in the larvae but did not see any difference (unpublished data). However, we reasoned that any potential increase in feeding response may not be readily detectable in the larvae because they feed continuously, already at a maximal rate. Therefore, we tested whether blocking synaptic transmission of *hug* neurons could suppress the feeding defect of *klu* mutants. We indeed observed a significant rescue of *klu* mutant feeding phenotype (Figure 6B).

We then carried out behavioral analysis on adults, since they are discontinuous feeders and thus may display an increased feeding behavior. Furthermore, one can visualize the quantity of food eaten by the size of the crop (Figure 6C). Experimental and control flies were placed in food vials containing standard fly food for several days. They were then transferred to yeast paste containing red dye. A striking result was observed. After 5 min, the experimental flies had a completely filled crop (Figure 6D, left column), whereas the control lines (Figure 6D, middle and right columns) had very little food in the crop. Even after 30 min, the control flies had very little food in their crops and only traces of red food were detectable in the midgut. By 180 min, both experimental and control flies showed the same degree of feeding. These results suggested that *hug* neurons are involved in regulating the initiation phase of feeding: control flies wait for a certain period before initiating feeding on the new food source, whereas decreasing *hug* neuronal signaling results in flies initiating their feeding immediately. When flies were transferred from yeast to colored yeast, or normal food to colored normal food, no difference was seen between experimental and control flies (unpublished data), indicating that *hug* neurons are not simply affecting the rate of feeding per se; we also did not observe a difference when transferring from yeast to normal food, indicating that the *hug* neuron-dependent behavioral effect is also not due to a simple fact of changing food sources. When flies were transferred from normal food into yeast containing 1M quinine (quinine is an aversive tastant), experimental flies again filled their crops earlier than controls (Figure S1). However, when flies were kept on yeast containing 1M quinine, and then transferred to yeast without the quinine, both experimental and control flies filled their crops with the new yeast within 5 min (Figure S1); analogously, when flies were starved before placing them on red yeast, both control and experimental flies filled their crops at about the same rate (Figure 6D, bottom row). These results suggest that the quality of previous food condition plays a role in defining *hug* neuronal function. Taken together, our studies support the view that *hug* neurons act within a neural circuitry in the brain that modulates feeding behavior based on chemosensory and nutrient signals.

## Discussion

### Central Relay of Gustatory Information

The identification of candidate chemosensory receptors in mammals and invertebrates has provided major insights into the molecular mechanisms underlying sensory information processing. In the *Drosophila* olfactory system, projections of OR-expressing sensory organs terminate at specific glomerular structures in the antennal lobe. The olfactory projection neurons then act in a second relay to convey the information to the mushroom bodies in the higher brain region. The gustatory organs, expressing specific GRs, project to a different brain region, the SOG, which has been implicated in gustatory signal transduction and feeding response in different insects. Our results indicate that the neurons that express the *hug* neuropeptide gene are likely candidates for acting as interneurons that transduce gustatory information. These comprise an assembly of about 20 neurons in the SOG. The close proximity of their dendrites with the axon terminals of gustatory sensory organs of the external head, and chemosensory organs of the internal pharynx, suggests a synaptic contact, but this requires functional verification. Whether the SOG is also organized into glomerular structure, like the antennal lobe, is not known. Such an organization has been suggested in adult *Drosophila* [17], although data on larvae have been lacking. Our results on the dendritic pattern of *hug* neurons also suggest a glomerular structure of the larval SOG, but this remains an open issue.

The *hug* neurons, in turn, send axons to at least three distinct targets: the ring gland, the pharyngeal muscles, and the protocerebrum. The projections to the ring gland and the pharyngeal muscles suggest that *hug* neurons coordinate sensory information with growth, metabolism, and food intake; the axon tracts to the protocerebrum suggest a role of *hug* neurons in transducing sensory signals for processing in the higher brain centers. These axon tracts are distinct from those of the olfactory projection neurons, projecting to a more dorsomedial region of the mushroom body, and adjacent to the median neurosecretory cells of the pars intercerebralis. Thus, *hug* neurons are ideally connected to undertake the role of integrating gustatory sensory signals with higher brain functions and feeding behavior.

### Chemosensory Adaptation, Nutrient Status, and Food Intake Response

The chemosensory systems of all animals play critical roles in modulating feeding behavioral response. Feeding behavior can have diverse aspects, including locating a food source, evaluating food for nutritional appropriateness, choosing between different food sources, and deciding to initiate or terminate feeding. Blocking synaptic transmission by tetanus toxin in the *hug* neurons alters a specific aspect of the feeding behavioral response. When transferred to a certain new food source, the control flies wait for a period before initiating feeding, whereas experimental flies start feeding almost immediately. In both cases, the size of the crop after a longer feeding period does not change, meaning that no difference is seen in the termination phase of feeding. It is interesting to note that GR66C1 (also named GR66a) neurons, which project to *hug* dendrites, have recently been shown to mediate aversive taste response [39,40]. This is consistent with the behavior of flies in which *hug* signaling is decreased, since they lose their “aversive” response, as manifested in the elimination of a wait period before feeding. This behavior is dependent on internal nutrient status, as well as food quality, since if animals are starved or given food with an aversive tastant beforehand (such as yeast with quinine), control flies also start feeding immediately on the new yeast source.

Insects have evolved a wide variety of feeding behaviors based on food identity, quality, and availability. Some of these are innate, whereas others are acquired through experience. For example, food preference in the tobacco hornworm is dependent on what they initially encounter after hatching. They are capable of growing on a wide variety of sources, but once they have fed on a particular food type, they will maintain this food preference [41,42]. In this context, a possible scenario is that *Drosophila* associate feeding with a particular food source with which they become familiar. When they encounter a different food source, they must first re-evaluate it, perhaps for nutrient content, or adapt to it, before initiating feeding. Therefore, *hug* neurons appear to regulate the decision to initiate feeding based on previous food experience.

### Central Integration of Feeding Behavior and Growth

In animals with a developed endocrine system, there is an intricate interdependence among feeding, growth, and neuroendocrine activity. *Drosophila* larvae are characterized by continuous feeding and a huge increase in organismal growth; in the adult, although no growth at organismal level takes place, a large cellular growth is required in the female for egg production. Both are highly dependent on feeding and the quality of food, such as protein content, and are under neuroendocrine control [2]. *klu* and *ppl* represent two genes that are required for food intake and growth in *Drosophila*. Mutations in both genes result in reduced food intake and growth. In addition, as young larvae, mutants display a wandering-like behavior, which is reminiscent of full-grown wild-type larvae, which stop feeding and move away from the food source just prior to pupariation, a process dependent on the neuroendocrine system [23]. Mutations in either of the genes lead to an upregulation of *hug* neuropeptide gene expression in the brain, whereas *hug* expression is downregulated in the absence of food signals.

**Figure 7:**
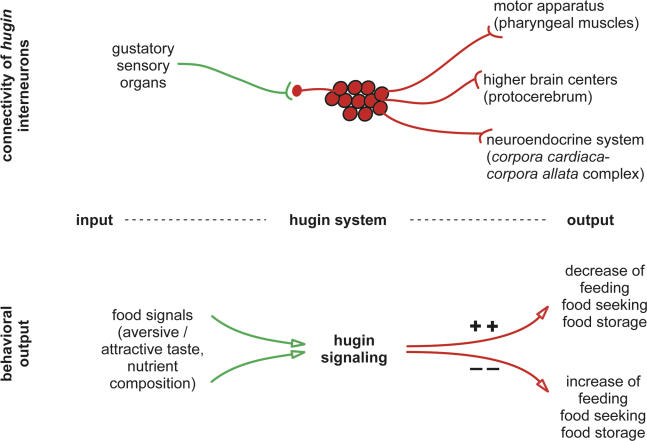
Model of *hug* as Modulator of Feeding Behavior The *hug* neurons, which express the neuropeptide gene *hug* and which interconnect gustatory sensillae via the SOG to the pharyngeal muscles, the protocerebrum, and the neuroendocrine organ, modulate chemosensory dependent feeding behavior. Increased *hug* signaling correlates with decreased feeding, whereas decreased *hug* signaling correlates with increased feeding (see Discussion section for details).

What could be the function of the *hug* neuropeptides? *hug* encodes at least two distinct neuropeptides [26]. One *(hug-*γ*)* has homology to an ecdysone triggering hormone, while the second (Drm-PK-2) is a pyrokinin with myostimulatory activity. *hug*-γ could be involved in controlling growth and metabolism. This view is supported by projection of *hug* neurons to the ring gland, the master neuroendocrine organ. In addition, overexpression of *hug* has been shown to cause molting defects [26]. Drm-PK-2, on the other hand, may play a role in modifying the mechanical aspect of food intake, which is supported by the projection of *hug* neurons to the pharyngeal muscles. One interesting possibility is that the different neuropeptides are translated or trafficked to different targets in subset of *hug* neurons. In this case, a common gene expression pattern can be utilized to send out different signals to the different targets, such as to the higher brain center, feeding apparatus, and neuroendocrine organ. This would be a mechanism for coordinating different growth-dependent processes with a common input signal, for example, from a particular food signal. In this context, one way to explain the upregulation of the *hug* gene in *klu* and *ppl* mutants would be that the level of *hug* gene differentially correlates with the degree of food-seeking response. High levels, as in the mutants that do not feed, would reflect lower feeding and food-seeking response, whereas low levels, as in the absence of food sensory input, would reflect increased food-seeking response. This would also be consistent with *hug* overexpression studies and with the correlation seen between decreasing *hug* neuronal activity and increased feeding (Figure 7). Further functional studies, including imaging analysis [43,44], should increase our understanding of how the *hug* neural circuit coordinates sensory perception, feeding behavior, and growth.

## Materials and Methods

### Feeding behavior assay

The larval feeding behavior assay was done as described previously [22]. Flies were allowed to lay eggs on apple juice agar plates containing colored yeast paste (150 mg Carmen Red, Sigma-Aldrich [St. Louis, Missouri, United States] per 100 g yeast paste). Given numbers of larvae from overnight egg collections were allowed to develop for 24 h at 25 °C and subsequently (2-h intervals) monitored for feeding and wandering phenotypes under a dissection microscope. For starvation experiments, wild-type larvae of late second instar were placed in petri dishes, containing filter paper that was soaked with either PBS (for complete starvation) or PBS containing 20% sucrose. For normal feeding conditions, fresh yeast paste was given. All feeding experiments were done at room temperature for 6 h. Overexpression studies were done using UAS-*hug* and tub-Gal4/TM3 fly lines [26], and heterozygote siblings were used as controls.

For larval rescue experiments, lines used were P(9036)/TM3 (parental line 1), UAS-TeTxLC; P(9036)/TM3 (parental line 2), and hugS3, P(9036)/TM3 (parental line 3). The genotypes assayed were +/UAS-TeTxLC; and P(9036)/P(9036) for control 1 (C1) + hugS3, P(9036)/P(9036) for control 2 (C2) and +/UAS-TeTxLC/+; hugS3, P(9036) for experimental (see Figure 6B). Feeding phenotypes were counted from 100 eggs per collection. Five independent collections per genotype were carried out.

For the adult feeding assay, experimental flies (*hug* promoter construct driving UAS-TeTxLC expression) and control flies (hugS3-Gal4 flies and UAS-TeTxLC flies crossed with wild-type) derived from 0- to 4-h egg collections were allowed to develop on indicated food for several days. After overnight feeding (for example, on standard fly food or PBS only), flies were allowed to feed on apple juice agar plates containing red-colored food being assayed. At given time points, randomly chosen individuals per genotype were removed and dissected under a dissection microscope. Preparations were fixed and mounted in Mowiol (see below) for microscopic analysis.

### Molecular and microarray analyses

The hug construct was made by cloning a 1.5-kb PCR fragment containing the *hug* regulatory region (amplified from genomic DNA using 5′– CTTCAGGGCCTTGGCTG and 5′– GGGACAACTGATGCCACG as primers) into a pCaSpeR-AUG-Gal4 vector [10]. The direct *hug* promoter YFP construct was made by replacing AUG-Gal4 of the *hug*-pCaSpeR construct, with a YFP fragment derived from YFP-pCS2+ vector (Clontech, Palo Alto, California, United States). Transgenic flies were obtained following standard injection protocols.

Microarray experiments were, in principle, done as described previously [45] using Affymetrix (Santa Clara, California, United States) GeneChips representing some 13,500 genes. Egg collections (0–4 h) of the P(9036)/TM3-GFP line were allowed to develop for an additional 22 h at 25 °C. Homozygous P(9036) larvae were hand-picked under a fluorescence microscope, and total RNA was isolated using the NucleoSpin RNA II Kit (Macherey-Nagel, Düren, Germany). GeneChip hybridization and data analysis was done as described [45].

### Histochemistry and fluorescence microscopy

Histochemical in situ hybridizations were done following standard protocols, with the slight modification of replacing the proteinase K digest with an overnight incubation of the dissected and fixed larval brains in methanol at −20 °C prior to hybridization. Samples were mounted either in Canada balsam or in Mowiol (12 ml glycerol, 4.8 g Mowiol 40–88, 12 ml H_2_O, and 24 ml 200 mM Tris [pH 8.5]), and images were taken using a Zeiss (Oberkochen, Germany) LSM 510 META in transmission mode. Fluorescence in situ hybridizations were done using the Tyramide Signal Amplification Kit (PerkinElmer, Wellesley, California, United States) and following the manufacturer's instructions. Overnight incubation with digoxygenin- and/or fluorescein-labeled riboprobes was followed by post-hybridization for additional 2 h. Detection of the first riboprobe with peroxidase (POD)-coupled antibody was performed by overnight incubation at 4 °C, followed by the first staining reaction using fluorescein-tyramide at 1:150 dilution and allowing the reaction to run for 10 min at room temperature. After inactivation of POD by incubation with 10 mM HCl in *Drosophila* Ringer's solution for 10 min, the second riboprobe was detected by overnight incubation with POD-coupled antibody at 4 °C. The second staining reaction was performed by applying Cy3-tyramide at 1:150 dilution for 10 min at room temperature. In cases of dual marker protein detection, primary antibodies (α–ßGal, Cappel, or α–GFP [Abcam, Cambridge, United Kingdom], used at 1:1,000) were applied together with first POD-antibody and secondary fluorescent antibody (Cy5-coupled α-rabbit, diluted at 1:200 [Jackson Immunoresearch, West Grove, Pennsylvania, United States]) was applied together with second POD-antibody. Samples were mounted in Mowiol and evaluated using a Zeiss LSM 510 META in confocal multitracking mode, generating optical 1- to 1.5-μm sections (using a Zeiss 40×/1.2W C-Apochromat lens) or 2.5-μm sections (using a Zeiss 25×/0.8Imm Plan-Neofluar lens). For direct detection and unmixing of GFP/YFP fluorescence, larval brains of appropriate genotype were dissected in chilled *Drosophila* Ringer's solution on ice, and mounted without fixation in PBS, using coverslips as spacers and nail polish as sealant. Immediate analysis was performed by emission fingerprinting, using a Zeiss LSM 510 META in confocal lambda mode. Other antibodies used for immunofluorescence were 22C10 diluted 1:100 (Developmental Studies Hybridoma Bank, Iowa City, Iowa, United States) and α–elav, diluted 1:300 (Developmental Studies Hybridoma Bank), as well as Alexa488-coupled α-rat and α-mouse antibodies, each diluted 1:200 (Molecular Probes, Eugene, Oregon, United States) and Cy3-coupled α-rat and α-rabbit antibodies diluted 1:200 (Jackson Immunoresearch, West Grove, Pennsylvania, United States). Nuclear counterstaining was performed using Draq5 (Biostatus Ltd., Leicestershire, United Kingdom), diluted 1:1,000 together with secondary antibodies. The GFP antibody co-labeling the corpora allata nuclei (Torrey Pines Biolabs, Houston, Texas, United States) was used at 1:1,000 dilution. The 3D reconstruction of optical sections and figure post-processing were done using Volocity 2.6 (Improvision, Lexington, Massachusetts, United States) and Photoshop 7.0 (Adobe Systems, San Jose, California, United States) on a Mac G4 computer (Apple Computer, Sunnyvale, California, United States).

## Supporting Information

Figure S1 Qualitative Graphical Representation of Feeding Analysis Flies were scored (none, traces, or full) based on amount of red food color in the gut and crop. Overnight treatment was for 12 h. Arrows represent transfer to fresh food, either of the same type or different. For each feeding regimen, two independent experiments were carried out, and for each experimental set and time point, ten individuals were taken out randomly and dissected. The top two graphs are graphical representations of the results depicted in Figure 6D. For experiments with quinine yeast as test food, 12 ± 2 individuals showed displayed phenotypes.
(3.1 MB TIF).

Figure S2 Fortuitous Staining of Corpora Allatum Fortuitous staining of corpora allatum nuclei (shown in green, arrows) by α-GFP antibody from Torrey Pines Biolabs in wild-type larvae (A) and adults (B and C) relative to the neuropile (22C10, shown in red) and the cortex (DNA marker Draq5, shown in blue). We do not know the reason for this, as α-GFP antibodies from other sources do not show this cross-reactivity.
(9.7 MB TIF).

## Abbreviations

CC/CA: corpora cardiaca/corpora allata
CNS: central nervous system
GFP: green fluorescent protein
GR: gustatory receptor
*hug*: *hugin*
*klu*: *klumpfuss*
OR: olfactory receptor
POD: peroxidase
*ppl*: *pumpless*
SOG: subesophageal ganglion
TH: tyrosine hydroxylase
VNC: ventral nerve cord
YFP: yellow fluorescent protein
